# Multidimensional Phenotypic and Microbiome Studies Uncover an Association Between Reduced Feed Efficiency in Sheep During Mycoplasmal Pneumonia and Microbial Crosstalk Within the Rumen-Lung Axis

**DOI:** 10.3390/vetsci12080741

**Published:** 2025-08-07

**Authors:** Lianjun Feng, Yukun Zhang, Xiaoxue Zhang, Fadi Li, Kai Huang, Deyin Zhang, Zongwu Ma, Chengqi Yan, Qi Zhang, Mengru Pu, Ziyue Xiao, Lei Gao, Changchun Lin, Weiwei Wu, Weimin Wang, Huibin Tian

**Affiliations:** 1 College of Pastoral Agriculture Science and Technology, Lanzhou University, Lanzhou 730020, China; 2 College of Animal Science and Technology, Gansu Agricultural University, Lanzhou 730046, China; 3 Institute of Animal Science, Xinjiang Academy of Animal Sciences, Urumqi 830000, China

**Keywords:** Mycoplasmal pneumonia of sheep, production performance, pulmonary microbiota, rumen microbiota, microbiota cross-talk, rumen-lung axis

## Abstract

Mycoplasmal pneumonia of sheep (MPS), caused by *Mesomycoplasma (Mycoplasma) ovipneumoniae*, profoundly impacts ovine productivity and survival. Although gut–lung microbiota interactions are increasingly recognized in respiratory diseases, whether similar crosstalk occurs between the lung and rumen microbiota in MPS-affected sheep remains unknown. To investigate alterations in the lung and rumen microbiota of sheep with MPS, the crosstalk between these microbial communities, and their impacts on growth phenotypes. From a cohort of 414 naturally infected six-month-old male Hu sheep, we selected 10 individuals with severe pulmonary pathology and 10 healthy controls for detailed phenotypic and microbiome analyses. Assessment of 359 phenotypic traits revealed that MPS significantly impairs feed efficiency and growth rate (*p* < 0.05). Through 16S rRNA gene sequencing, we found that MPS significantly altered the pulmonary microbiota community structure (*p* < 0.01), with a noticeable impact on the rumen microbiota composition (*p* = 0.059). *Succinivibrionaceae_UCG-001* was significantly depleted in both the rumen and lungs of diseased sheep (*p* < 0.05) and strongly associated with reduced average daily feed intake (*p* < 0.05). In addition, pulmonary *Pasteurella* and ruminal *Succinivibrionaceae_UCG-002* were significantly enriched in MPS-affected sheep, showed a strong positive correlation (*p* < 0.05), and were both negatively associated with feed efficiency (*p* < 0.05). Notably, *Pasteurella multocida* subsp. *gallicida* may act as a keystone species influencing feed efficiency. These findings point to a previously unrecognized rumen-lung microbial axis that may modulate host productivity in sheep affected by MPS. This work provides new insights into the pathogenesis of MPS and offers potential targets for therapeutic intervention and management.

## Simple Summary

This study sheds light on the impact of Mycoplasmal pneumonia of sheep (MPS) on sheep health and productivity, highlighting its economic consequences for the sheep industry. MPS, caused by *Mesomycoplasma (Mycoplasma) ovipneumoniae*, reduces lamb growth, lowers survival rates, and can lead to death, causing significant financial losses for farmers. Our research explored how MPS affects the microbial communities in the lungs and rumen, two key organs in sheep health. Using advanced genetic sequencing techniques, we identified specific microbial changes in these organs during MPS progression. Notably, we found that certain microbes in the lungs and rumen interact closely, potentially influencing immune responses and metabolic processes. These findings suggest that targeting these microbial interactions could help develop new treatments to reduce the severity of MPS and improve sheep health. Additionally, understanding these microbial dynamics may lead to better strategies for preventing and managing MPS, ultimately reducing economic losses in the sheep industry.

## 1. Introduction

Mycoplasmal pneumonia of sheep (MPS), also known as infectious pleuropneumonia of sheep, is a chronic respiratory infectious disease in sheep caused by *Mesomycoplasma (Mycoplasma) ovipneumoniae* (*M. ovipneumoniae*). Since its initial identification, this pathogen has been implicated in respiratory disease outbreaks across global sheep populations [1,2,3]. The MPS manifests with a range of symptoms following infection. While some sheep recover within a few weeks, severe cases may lead to acute fibrinous pneumonia, nodular pulmonary lesions, pulmonary abscesses, pleuritis, and even death [4]. Consequently, the disease impairs lamb growth and productivity, resulting in significant and often irreversible economic losses to the sheep industry annually [5]. However, the severity of the disease varies due to differences in strain virulence, host immune responses, and the presence of other complications, leading to diverse pathological outcomes [6]. The increasing prevalence of *M. ovipneumoniae*-associated pathology, exemplified by its identification as the primary etiological agent in severe pneumonia epizootics within the Norwegian muskox (*Ovibos moschatus*) Population in 2014 [7], has propelled this pathogen to the forefront of scientific inquiry. Research has elucidated several virulence mechanisms of *M. ovipneumoniae*, including modulation of macrophage activity, suppression of lymphocyte function, and production of hydrogen peroxide, which contributes to tissue damage [8,9,10]. For instance, some researchers have demonstrated that *M. ovipneumoniae* reduces the cytotoxicity of CD8 effector T cells, depletes regulatory T cells, and impairs normal cellular functions [11]. Previous study found that *M. ovipneumoniae* induces an inflammatory response in sheep airway epithelial cells via a Myeloid differentiation factor 88-dependent Toll-like receptor (TLR) signaling pathway [12]. Moreover, the *M. ovipneumoniae* can induce apoptosis in sheep bronchial epithelial cells via ROS-dependent JNK/p38 MAPK pathways and can induce sheep airway epithelial cell apoptosis through an Extracellular Signal-Regulated Kinase (ERK) signaling-mediated mitochondria pathway [13,14]. Despite these findings, current strategies for managing this disease are limited to prevention and control measures. There is a lack of effective therapeutic approaches and commercially available vaccines. This is partly attributable to the heterogeneity of *M. ovipneumoniae* and the limited understanding of its pathogenic mechanisms [6].

The pulmonary microbiota plays a critical role in maintaining lung health and serves as a key component of the immune system in protecting the lungs from harmful inflammation [15]. It is involved in the normal development of the respiratory tract, regulation of respiratory immunity, and prevention of pathogen colonization, thereby contributing to overall respiratory health [16]. The composition of the human pulmonary microbiota is dynamically influenced by pulmonary diseases. In patients with asthma and chronic obstructive pulmonary disease (COPD), an increased abundance of pathogenic Proteobacteria, particularly *Haemophilus*, has been observed [17,18]. Patients with interstitial pneumonia exhibit a decrease in *Firmicutes* and *Streptococci*, accompanied by an increased abundance of *Prevotella* and *Veillonella* [19]. Pulmonary diseases also alter the lung microbiota in ruminants. For example, *Mycoplasma* and *Pasteurellaceae* exhibit higher abundance and frequency in lung tissue samples from calves displaying signs of bovine respiratory disease (BRD) [20]. In sheep, pneumonia significantly reduces lung microbial diversity and alters taxonomic composition [21]. In humans, the stability of the gut microbiota is crucial for maintaining lung health. The gut-lung axis represents a significant pathway through which the gut microbiota influences pulmonary diseases. Dysbiosis of the gut microbiota can trigger a variety of pulmonary diseases through the gut-lung axis, including viral pneumonia, asthma, tuberculosis, and COPD [22,23]. Mechanistically, the gut microbiota confers protection against lipopolysaccharide (LPS)-induced acute lung injury (ALI) by regulating the TLR4/NF-κB signaling pathway [24,25,26]. In ruminants, the rumen microbiota is a pivotal component of the digestive system, converting plant fiber into absorbable nutrients and providing 70% to 80% of the animal’s energy [27]. Beyond digestion, the rumen microbiota influences feed conversion efficiency, methane production, and milk yield in dairy cows [28]. It is also associated with diseases such as bovine mastitis [29] and postpartum ketosis [30]. For instance, inulin alleviates inflammatory responses in subclinical mastitis by modulating microbial communities and metabolites [31]. Additionally, alterations in rumen microbiota structure have been observed in Holstein heifers with high-altitude pulmonary hypertension [32]. However, the alterations in pulmonary and ruminal microbiota in *M. ovipneumoniae*-infected sheep, their potential interrelationship, and whether they contribute to the impact of MPS on host productivity remain unclear.

To explore the effects of MPS on the lung and rumen microbiota of sheep, the crosstalk between these two microbial communities, and whether they are involved in mediating the impact of MPS on sheep growth phenotypes. In this study, we comprehensively evaluated the effects of MPS on 19 productivity-related phenotypes, including growth performance and feed efficiency, to quantify the disease’s impact on sheep productivity. Using 16S rRNA gene sequencing, we characterized microbial community structures in the lungs and rumen of both diseased and healthy sheep. Furthermore, we examined potential crosstalk between these microbial communities to elucidate whether alterations in one compartment influence the composition or function of the other.

## 2. Materials and Methods

### 2.1. Animal and Management

A total of 414 male Hu lambs, with consistent genetic backgrounds and born during the same period, were randomly selected from the same commercial sheep farm. On the weaning day, they were transported by road to the Minqin Experimental Base of Lanzhou University (38°43′41′′ N, 103°13′ E) to establish a cohort for natural infection. These sheep were infected with Mycoplasma pneumonia under natural conditions without human intervention. During the experiment, all sheep were housed in individual pens until they reached the commercial slaughter age of 6 months (180 days) and were not treated with any antibiotics or probiotics. Throughout the rearing period, all sheep were managed under standardized conditions. In accordance with the recommended feeding standards for Chinese sheep (NY/T816-2004), the sheep were fed a uniform diet (Supplementary Table S1). Fresh water was provided ad libitum to ensure unrestricted access to drinking water. Body weight (BW), chest circumference (measured as the circumference around the chest from the back end of the scapula), and feed intake were recorded every 20 days from 80 to 180 days of age, in accordance with the Technical Specification for the Measurement of Breeding Sheep Production Performance (NY/T1236-2023). All sheep were subjected to the commercial slaughter process at 180 days of age following a 12 h fasting period.

Average daily gain (ADG) was calculated as: (BW at t_2_ − BW at t_1_)/(t_2_ − t_1_). Average daily feed intake (ADFI) was the total feed intake from t_1_ to t_2_ divided by the time interval (t_2_ − t_1_). Metabolic body weight (MBW) was calculated as BW^0.75^. Feed conversion ratio (FCR) was calculated as the ratio of ADFI to ADG (ADFI/ADG). The Kleiber ratio (KR) was calculated as the ratio of ADG to MBW (ADG/MBW). Gross feed efficiency (GFE) was calculated as the ratio of ADG to ADFI (ADG/ADFI). Dry matter intake per unit metabolic body weight (DMIMBW) was calculated as the ratio of ADFI to MBW (ADFI/MBW). Residual gain (RG) was determined by regressing total weight gain against initial BW and cumulative feed intake, with the residuals from this regression model representing the portion of growth performance not explained by baseline body mass or feed consumption. The relative growth rate (RGR) was calculated using the formula: RGR = (lnBW_2_ − lnBW_1_)/(t_2_ − t_1_), where BW_1_ and BW_2_ represent the initial and final BW, respectively, and t_1_ and t_2_ represent the corresponding time points.

### 2.2. Blood Sample Collection and Hematological Measurements

At 180 days of age, whole blood samples were collected from the jugular vein of all 414 sheep using 2 mL anticoagulant tubes, following a 12 h fasting period. Prior to sample collection, the ProCyte Dx* fully automated five-part differential animal blood cell analyzer (ProCyte Dx, IDEXX, Westbrook, ME, USA) was cleaned and calibrated, with the analysis mode set to “sheep”. Blood samples were collected and processed within 30 min to prevent blood cell sedimentation, with gentle inversion performed periodically. Hematological parameters were measured using the ProCyte Dx* analyzer. The following parameters were recorded: red blood cell (RBC) count, hemoglobin (HGB) concentration, hematocrit (HCT), mean corpuscular volume (MCV), mean corpuscular hemoglobin (MCH), mean corpuscular hemoglobin concentration (MCHC), red cell distribution width (RDW_SD and RDW_CV), reticulocyte count (RET1) and percentage (RET2), reticulocyte hemoglobin content (RET_He), white blood cell (WBC) count, neutrophil (NEUT1), lymphocyte (LYMPH1), monocyte (MONO1), eosinophil (EO1), and basophil (BASO1) counts, neutrophil (NEUT2), lymphocyte (LYMPH2), monocyte (MONO2), eosinophil (EO2), and basophil (BASO2) percentages, platelet (PLT) count, mean platelet volume (MPV), and plateletcrit (PCT). Raw data were exported and managed using Excel 2019. Outliers were identified and excluded using a customized R script.

### 2.3. Sample Collection

To ensure standardized sample collection and minimize operator bias, a partitioned dual-operator approach was employed. Two licensed veterinarians, each holding valid veterinary certificates, were assigned specific roles: one veterinarian was responsible for isolating and collecting lung samples, while the second veterinarian focused on rumen sample collection. A third-party quality inspector supervised the entire procedure to ensure adherence to protocols and track procedural timing.

Immediately post-slaughter, the first veterinarian rapidly excised the lungs and placed them into designated sterile surgical trays. The lungs were then subjected to a “ morphological-touch dual-modal assessment “ for preliminary pathological evaluation. This assessment combined visual inspection (surface color, vascular distribution, lesion morphology) with tactile evaluation (tissue elasticity, texture) to identify suspicious lesions. Any consolidated area with a diameter exceeding 5 mm was marked as a potential lesion and classified as diseased tissue. Pulmonary lymph nodes were carefully separated using surgical scissors, weighed to an accuracy of 0.01 g, and consolidated tissue samples were collected for histopathological slide preparation. After being removed from the thoracic cavity, the lungs were immediately placed in a clean area for lavage. Sterile PBS was dropped into the lungs. After gently kneading the lungs for 2 min, the liquid was recovered. Within 30 min post-slaughter, approximately 45–50 mL of PBS lavage fluid was collected from each lung. A blank control, consisting of PBS from the same batch subjected to identical handling but not used for lavage, was included. The PBS lavage mixture was centrifuged at 13,000 rpm for 30 min at 4 °C. The lavage pellet was subsequently transferred to a 2 mL sterile tube and stored at –80 °C until DNA extraction for subsequent analysis. The second veterinarian collected liquid from the rumen’s ventral sac using a 50 mL syringe. The collected liquid was filtered through four layers of sterile gauze to remove particulate matter and aliquoted into sterile centrifuge tubes (5 mL per tube). Samples were immediately frozen in liquid nitrogen and stored at −80 °C for subsequent microbial community analysis.

### 2.4. Histological Analysis

Fresh lung tissue samples were obtained from the cranial lobe of the right lung, which was accessible in all animals. Tissue sections of approximately 1 cm^3^ were excised using sterile instruments. These samples were fixed in 4% paraformaldehyde for 24 h, followed by paraffin embedding and sectioning into 5-μm-thick slices. Sections underwent dewaxing through sequential immersion in xylene (15 min × 2), absolute ethanol (5 min × 2), 95% ethanol (5 min × 2), and 80% ethanol (5 min × 2). Following dewaxing, sections were stained with hematoxylin for 5 min, rinsed with water for 10 min, differentiated in 1% hydrochloric acid alcohol for 30 s, and washed in distilled water for 30 s. Sections were then stained with 0.5% eosin for 3 min, followed by a 30 s wash in distilled water. Dehydration was performed through sequential immersion in 80% ethanol (5 min × 2), 95% ethanol (5 min × 2), absolute ethanol (5 min × 2), and xylene (15 min × 2). Sections were mounted with neutral resin and observed under a light microscope. Histopathological assessment of lung sections was conducted using a semi-quantitative scoring system adapted from Li et al. [33]. The following parameters were evaluated: exudates, hyperemia, congestion, neutrophilic infiltrates, intra-alveolar hemorrhage, debris, and cellular hyperplasia. Each parameter was scored on a scale of 0 to 3 (0 = absent, 1 = mild, 2 = moderate, 3 = severe). Final scores were summed and subjected to statistical analysis.

### 2.5. Animal Grouping

In this study, a total of 414 sheep underwent pulmonary morphological assessment, lung weight measurement, and hematological testing conducted by a licensed veterinarian. Additionally, the severity of lesions was determined through histopathological analysis. Based on these evaluations, 34 sheep were initially identified as having Mycoplasma pneumonia, resulting in a prevalence rate of 9%. Subsequently, from the 34 infected sheep, a subset of 10 sheep with the highest disease severity, determined by lymph node weight and pathological scoring, was selected for the disease group. To ensure sample balance, a control group of 10 healthy sheep was selected from those with no pulmonary lesions, all of which had a pathological score of 0 and the lowest lymph node weights. For further investigation, the pulmonary microbiome of these 20 sheep was analyzed using 16S rRNA gene sequencing to assess the status of Mycoplasma pneumonia. All subsequent analyses in this study were based on these 20 sheep.

### 2.6. Sequencing and Analysis of 16S rRNA Gene

Microbial genomic DNA was extracted from lung lavage pellet and rumen content samples using the QIAamp Fast DNA Stool Mini Kit (TransGen Biotech, EE301-01, Beijing, China) following the manufacturer’s instructions. The V3-V4 region of the 16S rRNA gene was amplified using primers 341F (CCTAYGGGRBGCASCAG) and 806R (GGACTACNNGGGTATCTAAT). PCR amplification system (total 50 μL): 10 μL of 5×PrimerStar buffer, 2 μL of dNTPs, 2 μL of 341F primer, 2 μL of 806R primer, 2 μL of DNA template, 1 μL of PrimerStar polymerase, and 31 μL of sterile ddH_2_O. PCR amplification program: pre-denaturation at 95 °C for 10 min; 30 cycles of denaturation at 95 °C for 30 s, annealing and renaturation at 52 °C for 30 s, and extension at 72 °C for 30 s; final extension at 72 °C for 10 min; electrophoresis to detect the amplification results. Amplicons were sequenced on the NovaSeq PE250 platform (Illumina, Novogene Biotech Co., Ltd., Beijing, China), yielding 20 lung and 20 rumen microbial samples. Paired-end sequencing reads were merged using VSEARCH (v2.7.1+52) to generate complete reads, followed by primer trimming and quality control. The key filtering criterion was set using --fastq_maxee_rate 0.01. The total number of high-quality reads retained across all samples after filtering was 3,105,865; the average length of reads was 442.3 (range: 44 to 484). Low-abundant sequences as amplicon sequence variants (ASVs) with a relative abundance of less than 0.01% across all samples, and those that were detected in fewer than 3 samples, and further denoising was performed using USEARCH (v10.0.240) to identify high-confidence ASVs. A feature table containing ASV abundance information was generated using USEARCH, and taxonomic annotation was performed using the SILVA (v138) database. Rarefaction normalization was conducted using the *Vegan* (v2.7-1) R package to account for sequencing depth variations, and alpha diversity indices were calculated to assess microbial richness and evenness. Beta diversity was analyzed using USEARCH to evaluate structural differences in microbial communities across samples. The entire analysis adopted the 16S rRNA amplicon analysis method by Liu Yongxin et al. [34].

### 2.7. Statistical Analysis

Statistical analysis and data visualization were performed using R (v4.3) and GraphPad Prism (v9.5). Comparison of productivity and clinical phenotypic measurement data between groups was performed using a Kruskal–Wallis method (between two groups). Differential microbial analysis was performed using the edgeR method based on the ASV abundance feature table [35], with a significance threshold of *p* < 0.05 to identify differentially abundant microbial taxa. Spearman’s rank-order correlation was used for statistical correlation analysis of differential microbial taxa between the two organs, with *p* < 0.05 indicating a significant correlation. In all statistical tests, *p* < 0.01 was considered highly significant, *p* < 0.05 was considered statistically significant, and 0.05 < *p* < 0.1 was considered marginally significant.

## 3. Results

### 3.1. Statistical Comparison of Gross Lung Lesions, Pulmonary Lymph Node Weights, and Histopathological Scores Between Diseased and Healthy Groups

**Figure 1:**
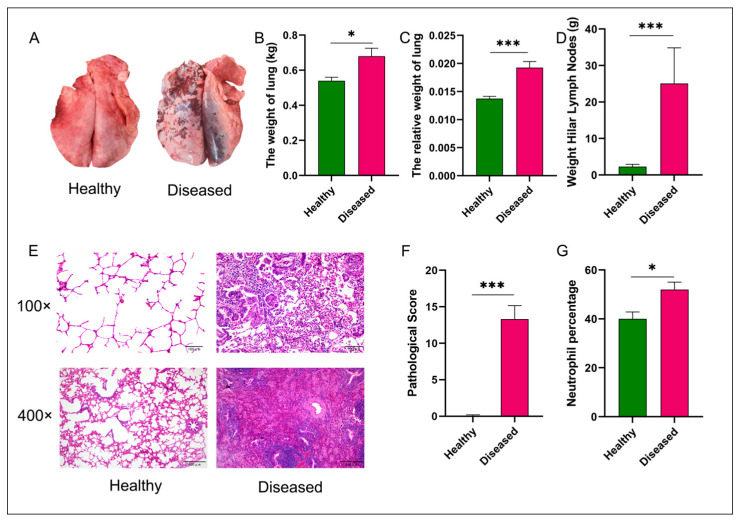
Comparative analysis of pulmonary phenotypes and histopathology between healthy and diseased groups. Panel (**A**) displays representative images of gross pulmonary morphology, illustrating distinct differences between healthy and diseased lungs. Panels (**B**,**C**) provide quantitative data on absolute and relative lung weights, respectively, with significant increases observed in the diseased group. Panel (**D**) details the weight of hilar lymph nodes, further emphasizing the pathological changes in diseased animals. Histological analysis in panel (**E**) shows H&E-stained sections at 100× and 400× magnifications, revealing structural alterations and inflammatory infiltrates in diseased lungs. Panel (**F**) quantifies alveolitis and fibrosis pathological scores, demonstrating significantly higher scores in diseased lungs. Lastly, panel (**G**) reports the percentage of neutrophils in blood samples, indicating an elevated inflammatory response in the diseased group. Statistical significance was assessed using a Kruskal–Wallis test, with * *p* < 0.05 and *** *p* < 0.001 denoting significant differences between groups.

We utilized a multidimensional pathological assessment system, encompassing morphological evaluation, weight measurement, histopathological analysis, and hematological examination, to characterize the extensive and distinctive pathological changes in the lungs of sheep affected by MPS. Morphological evaluation revealed that healthy control lungs displayed typical pink coloration with uniform vascular distribution, whereas diseased lungs exhibited marked dark consolidation lesions and vascular network disruption (Figure 1A). The hilar lymph nodes and lung absolute weights were significantly elevated in the MPS group compared to the healthy group (0.68 kg vs. 0.54 kg, *p* < 0.05), with a highly significant increase in the relative weight of lung (0.02 vs. 0.01, *p* < 0.01; Figure 1B–D). Histopathological analysis at 100× and 400× magnification demonstrated preserved alveolar architecture in healthy lungs, while diseased lungs showed pronounced neutrophilic infiltration within alveolar spaces and lymphocytic/plasmacytic infiltration in interstitial regions (Figure 1E). These histological alterations were corroborated by significantly higher pathological scores in the diseased group (*p* < 0.01; Figure 1F). Hematological analysis further revealed a significant increase in peripheral blood Neutrophil percentage (NP, 2.29% vs. 25.08%, *p* < 0.05) (Figure 1G), indicative of an inflammatory response associated with MPS. Collectively, these findings underscore the characteristic pulmonary pathology of MPS and validate the robustness of our experimental design.

### 3.2. Growth Performance and Feed Efficiency of Hu Sheep Affected by MPS

**Figure 2:**
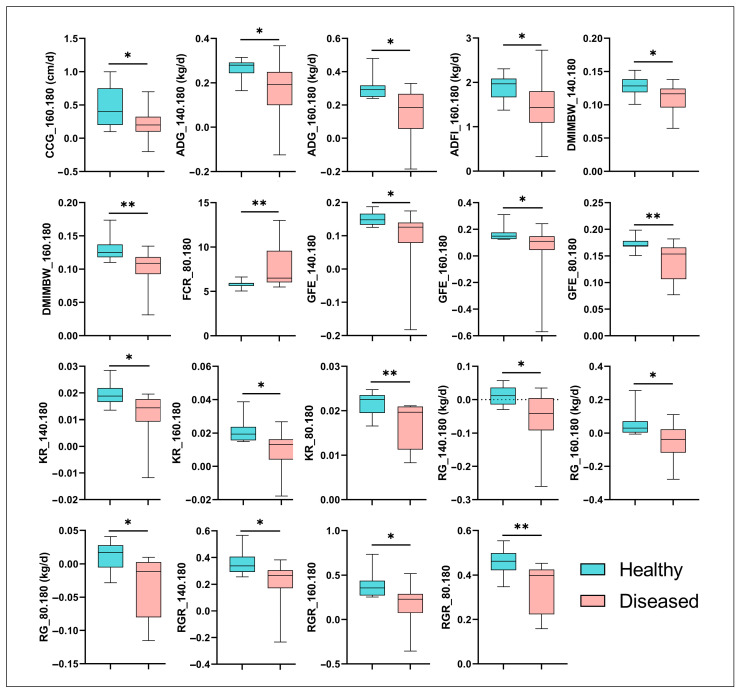
Growth performance and feed efficiency of hu sheep affected by MPS. CCG: Chest circumference gain; ADG: Average daily gain; ADFI: Average daily feed intake; DMIMBW: Dry matter intake per unit metabolic body weight; FCR: Feed conservation rate; GFE: Gross feed efficiency; KR: Kleiber ratio; RG: Residual gain; RGR: Relative growth rate. Statistical significance was determined using Kruskal–Wallis test; * *p* < 0.05, ** *p* < 0.01.

To investigate the impact of MPS on the productivity of sheep, we compared growth and feed efficiency traits between healthy and diseased animals. Our results demonstrated that ADG, DMIMBW, GFE, KR, RGR, and RG were significantly reduced in the diseased group compared to the healthy group from 140 to 180 days of age (*p* < 0.05). Notably, the DMIMBW, GFE, KR, and RGR exhibited highly significant reductions in the diseased group from 160 to 180 days of age (*p* < 0.01). Additionally, the GFE, KR, RG, and RGR remained consistently and significantly lower in the diseased group across the entire age range of 80 to 180 days (*p* < 0.05). Furthermore, the ADFI and the coefficient of chest circumference gain (CCG) were significantly decreased in the diseased group (*p* < 0.05, Figure 2). Collectively, these findings indicate that MPS exerts a significant negative impact on the productivity of Hu sheep, underscoring the importance of addressing this disease to improve livestock performance.

### 3.3. Identification of Mesomycoplasma Ovipneumoniae as a Key Pathogen in MPS via 16S rRNA Gene Sequencing

The sequencing depth was sufficient and consistent across samples, as confirmed by the dilution curves (Figure S1). A total of 3,105,865 effective reads were obtained, and after annotation, 24 phyla, 42 classes, 109 orders, 204 families, 402 genera, and 8457 ASVs were identified. Of these ASVs, 110 were affiliated with the genus *Mycoplasma* (Supplementary Table S2). Specifically, fifteen ASVs were annotated only to the genus level, while 18 ASVs were annotated to *s_Mesomycoplasma_ovipneumoniae*, 23 to *s_Mycoplasmopsis_arginini*, 17 to *s_Mycoplasmopsis_bovigenitalium*, and 11 to *s_Mycoplasmopsis_hyopharyngis*. The remaining ASVs were annotated to *s_uncultured_Mycoplasma_sp.* (9 ASVs), *s_Mycoplasmopsis_californica* (4 ASVs), *s_Mycoplasma_californicum_HAZ160_1* (3 ASVs), *s_Mycoplasma_ovis_str._Michigan* (3 ASVs), *s_Metamycoplasma_gateae* (2 ASVs), *s_Mycoplasma_simbae* (2 ASVs), *s_Mycoplasmopsis_adleri* (2 ASVs), and *s_Mycoplasma_indiense* (1 ASV). All ASVs affiliated with *Mesomycoplasma_ovipneumoniae* were exclusively detected in lung samples. At the species level, *Mesomycoplasma_ovipneumoniae* was detected in all diseased lung samples, with an average abundance of 7.73%. Notably, it was also present in the lungs of 7 healthy sheep, albeit at a much lower average abundance of 0.07% (*p* < 0.01). Notably, *Mesomycoplasma_ovipneumoniae* is the only *Mycoplasma* taxon that was present in all diseased lung samples and exhibited a significant difference in abundance compared to healthy lungs. This finding underscores the potential role of *Mesomycoplasma_ovipneumoniae* as a key pathogen in MPS and highlights its presence even in asymptomatic hosts.

### 3.4. Comparative Analysis of Pulmonary and Rumen Microbiota Diversity in Healthy and MPS-Sheep

**Figure 3:**
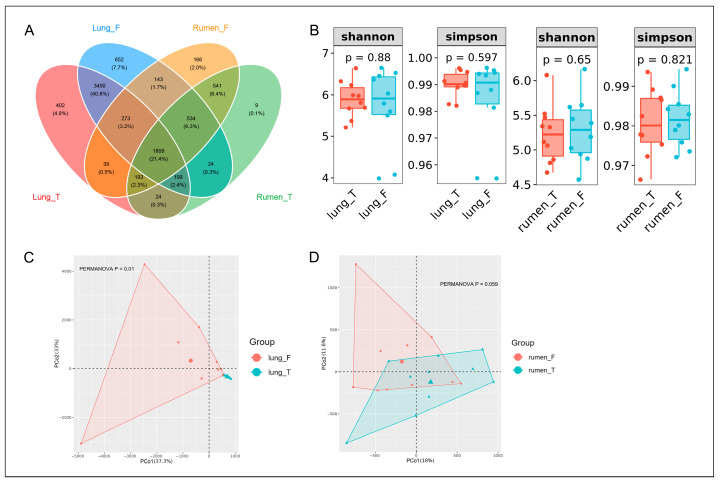
Microbial community diversity and structural differences between experimental groups. Panel (**A**) displays representative Venn diagram comparing ASVs shared across microbiota from lung_T, lung_F, rumen_T, and rumen_F groups. Panel (**B**) displays representative comparison of alpha diversity metrics for the lung and rumen microbiota between healthy and diseased groups. Panels (**C**,**D**) provide Principal Coordinates Analysis (PCoA) of the pulmonary and rumen microbiota.

We utilized Venn diagrams to compare shared and unique ASVs across the four groups (Figure 3A). The analysis revealed that all groups shared 1808 ASVs. Diseased lungs (lung_F) and diseased rumen (rumen_F) shared 2758 ASVs, while healthy lungs (lung_T) and healthy rumen (rumen_T) shared 2224 ASVs. The healthy lungs and rumen had 402 and 9 unique ASVs, respectively, whereas diseased lungs and rumen had 652 and 166 unique ASVs, respectively. Among the 652 unique ASVs in diseased lungs (lung_F) (Supplementary Table S3), 31 were affiliated with the genus *Mycoplasma*, including 2 ASVs of *s_Mesomycoplasma_ovipneumoniae*, 8 ASVs of *_Mycoplasma*, 2 ASVs of *s_Mycoplasma_simbae*, 6 ASVs of *s_Mycoplasmopsis_arginini*, 1 ASV (ASV_5858) of *s_Mycoplasmopsis_bovigenitalium*, 1 ASV of *s_Mycoplasmopsis_californica*, and 11 ASVs of *s_Mycoplasmopsis_hyopharyngis*. *Mesomycoplasma_ovipneumoniae* primarily infects sheep and is a well-documented pathogen in ovine pneumonia. *Mycoplasma_simbae* is known to predominantly infect cattle, while *Mycoplasmopsis_arginini* has a broader host range, including goats, sheep, cats, camels, and humans. *Mycoplasmopsis_bovigenitalium*, *Mycoplasmopsis_californica*, and *Mycoplasmopsis_hyopharyngis* are primarily associated with cattle infections.

To further elucidate the pulmonary microbial community structure changes and the subsequent alterations in rumen microbiota structure during MPS, we initially compared the microbial diversity of the two organs between healthy and diseased sheep. Alpha diversity analysis showed non-significant differences in Shannon and Simpson indices in diseased lungs versus healthy lungs, with no significant differences in ruminal α-diversity (Figure 3B). Beta diversity analysis via Bray–Curtis dissimilarity demonstrated clear separation between healthy and diseased lungs, and weaker separation between healthy and diseased rumens. Permutational multivariate analysis of variance confirmed significant differences in pulmonary microbiota structure (*p* < 0.05) and marginally significant differences in rumen microbiota (*p* = 0.059), underscoring MPS-induced microbial restructuring (Figure 3C,D). These findings highlight the distinct impact of MPS on pulmonary and ruminal microbiota and composition.

### 3.5. Taxonomic Composition of Lung and Rumen Microbiota in Healthy vs. Diseased Groups

**Figure 4:**
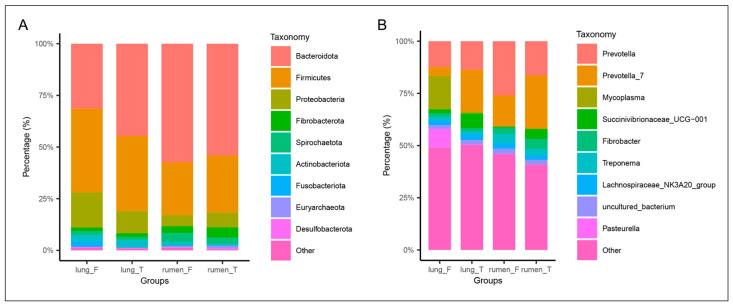
Comparison of lung and rumen microbiota composition between healthy and diseased groups. Panel (**A**) displays representative relative abundance of microbial taxa at the phylum level in lung_T, lung_F, rumen_T, and rumen_F groups. Panel (**B**) displays representative relative abundance of microbial taxa at the genus level in lung_T, lung_F, rumen_T, and rumen_F groups.

To investigate the changes in lung and rumen microbiota composition following MPS, comparisons were made at both the phylum and genus levels. In healthy sheep, the lung microbiota was primarily composed of *Bacteroidetes*, *Firmicutes*, *Proteobacteria*, and *Actinobacteria*. In contrast, sheep with Mycoplasmal pneumonia exhibited a significant increase in the relative abundance of *Proteobacteria* and *Fusobacteriota* in the lung microbiota (Figure 4A). In the rumen, a slight increase in the relative abundance of *Spirochaetes* and *Actinobacteria* was observed in diseased sheep compared to healthy sheep. At the genus level, we observed a significant enrichment of *Mycoplasma* and *Pasteurella* in the lungs of diseased sheep, where they constituted a dominant proportion of the overall pulmonary microbiota. However, the abundance of *Prevotella_7* and *Succinivibrionaceae_UCG-001* decreased, a trend mirrored by decreased *Succinivibrionaceae_UCG-001* in the rumen of diseased sheep (Figure 4B).

### 3.6. Differential Microbial Analysis of Lung and Rumen Between Healthy and MPS-Sheep

**Figure 5:**
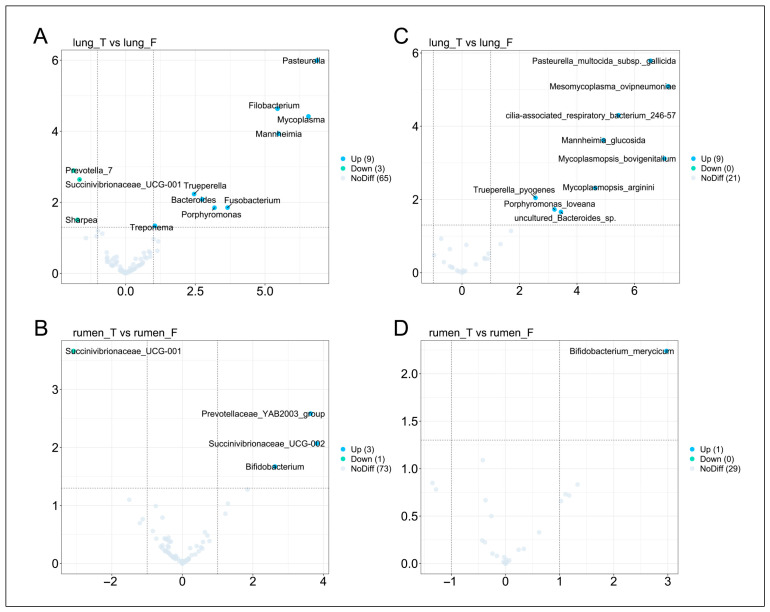
Differential microbial analysis between healthy and diseased groups in lung and rumen. Panels (**A**,**B**) display representative differentially abundant lung microbial genera and rumen microbial genera between healthy and diseased groups. Panels (**C**,**D**) provide differentially abundant lung microbial species and rumen microbial species between healthy and diseased groups.

To identify key microbial taxa in MPS, we performed differential abundance analysis. In the diseased sheep, nine genera were significantly enriched in the lungs: *Pasteurella*, *Filobacterium*, *Mycoplasma*, *Mannheimia*, *Trueperella*, *Bacteroides*, *Fusobacterium*, *Porphyromonas*, and *Treponema*. Conversely, *Prevotella_7*, *Succinivibrionaceae_UCG-001*, and *Sharpea* were significantly depleted (Figure 5A). Ruminal analysis revealed three enriched genera in diseased sheep: *Prevotellaceae_YAB2003_group*, *Succinivibrionaceae_UCG-002*, and *Bifidobacterium*, while *Succinivibrionaceae_UCG-001*, also depleted in the lung, was similarly reduced in the rumen (Figure 5B). At the species level, nine taxa were significantly enriched in the lung, including three *Mycoplasma* species: *Mesomycoplasma_ovipneumoniae*, *Mycoplasmopsis_bovigenitalium*, and *Mycoplasmopsis_arginini*. The remaining six enriched species were *Pasteurella_multocida_subsp._gallicida*, *cilia-associated_respiratory_bacterium_246-57*, *Mannheimia_glucosida*, *Trueperella_pyogenes*, *Porphyromonas_loveana*, *uncultured_Bacteroides_sp* (Figure 5C). Only one species, *Bifidobacterium_merycicum*, was significantly enriched in the rumen of diseased sheep (Figure 5D).

### 3.7. Correlation Analysis Between Differentially Abundant Lung Microbes, Differentially Abundant Rumen Microbes, and Growth Performance/Feed Efficiency

**Figure 6:**
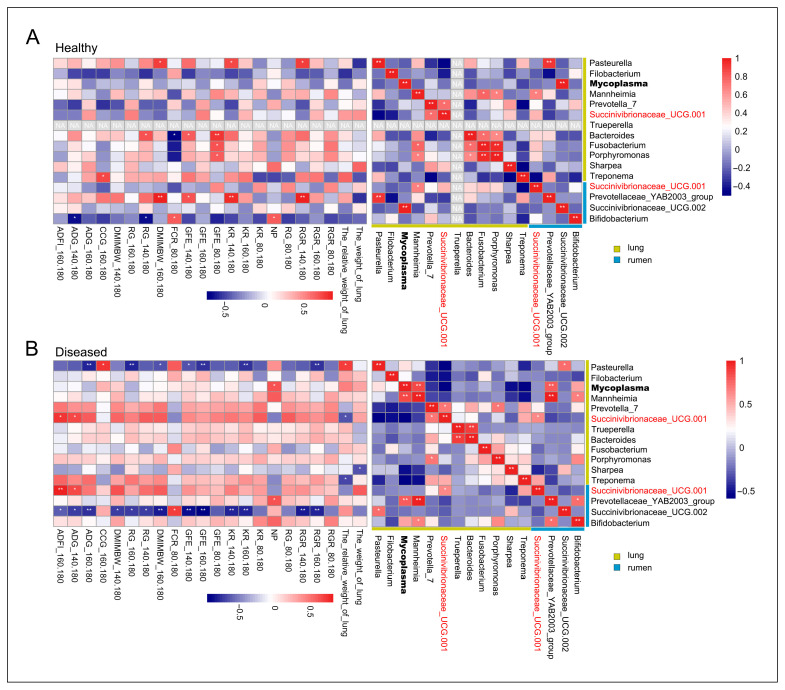
Heatmap of the correlation analysis between differential microbiota in the lung and rumen and growth performance and feed efficiency. Panel (**A**) displays representative heatmap of correlations between differentially abundant microbial genera in the lung and rumen, and growth performance and feed efficiency traits in healthy sheep. Panel (**B**) displays representative heatmap of correlations between differentially abundant microbial genera in the lung and rumen, and growth performance and feed efficiency traits in diseased sheep. Microbes shown in red font represent differential taxa present in both the lungs and the rumen. The method of correlation analysis uses Pearson; * *p* < 0.05, ** *p* < 0.01.

In the healthy sheep, pulmonary *Mycoplasma* exhibited strongly significant positive correlations with ruminal *Succinivibrionaceae UCG-002* (r = 0.99, *p* < 0.01). *Pasteurella* showed a strong positive correlation with the rumen taxon *Prevotellaceae_YAB2003_group* (r = 0.92, *p* < 0.05) (Figure 6A). In the diseased sheep, a strong positive correlation was observed between pulmonary *Mycoplasma*/*Mannheimia* and the rumen taxa *Prevotellaceae_YAB2003_group* (*p* < 0.01). Additionally, *Mycoplasma* exhibited strongly significant positive correlations with *Mannheimia* in the lungs (r = 0.86, *p* < 0.01). *Pasteurella* showed a strong positive correlation with the rumen taxon *Succinivibrionaceae UCG-002* (r = 0.72, *p* < 0.05). Notably, *Succinivibrionaceae UCG-001*, a taxon found in both the lung and rumen, exhibited a significant positive correlation between its lung and rumen populations (r = 0.63, *p* < 0.05) (Figure 6B). This finding further suggests an interaction between the pulmonary and ruminal microbiota.

In the diseased sheep, correlation analysis between differentially abundant microbes and phenotype revealed that pulmonary *Mycoplasma* was significantly positively correlated with NP (r = 0.64, *p* < 0.05). Notably, the abundance of *Succinivibrionaceae_UCG-001* in both the lung and rumen was significantly positively correlated with ADFI during the 160–180 days (*p* < 0.05). *Pasteurella* was negatively correlated with ADG, GFE, KR, RG, and RGR in 160–180 days (*p* < 0.01), and ruminal *Succinivibrionaceae UCG-002* was negatively correlated with ADG, ADFI, DMIMBW, GFE, KR, RG, and RGR in 140–180 and 160–180 days (*p* < 0.05) (Figure 6A). These results implicate *Pasteurella* and ruminal *Succinivibrionaceae UCG-002* as the key driver of reduced growth performance and feed efficiency in MPS. Additionally, pulmonary *Pasteurella_multocida_subsp._gallicida* exhibited strong negative correlations with ADG, GFE, KR, RG, and RGR in 160–180 days (*p* < 0.01) (Figure S2).

## 4. Discussion

The *M. ovipneumoniae*-induced pneumonia in sheep can affect all age groups, with lambs being the most susceptible. This pathogen displays cross-species transmission potential, infecting not only ovine but also caprine and cervid. Furthermore, the detrimental effects of pneumonia on growth performance and feed efficiency in swine, horn growth rate in bighorn sheep, and average daily gain and milk yield in dairy cattle are well-established [36,37,38,39]. Our findings demonstrate a significant impact of MPS on growth performance and feed conversion ratio in Hu sheep. Given that sample collection and pathological phenotyping occurred at 180 days, this observation supports the hypothesis that MPS exerts its influence on these parameters during the 140–180 days and 160–180 days intervals. This study reinforces existing literature, underscoring the critical need for efficacious therapeutic and prophylactic strategies against MPS. Consequently, further investigation into the pathogenesis and underlying molecular mechanisms of MPS is warranted. The significance of gut microbiota-host interactions has been increasingly recognized in recent years. The paradigm of a “gut-target organ axis”, wherein gut microbiota can modulate the pathology and physiology of distal organs, has gained widespread acceptance. In the context of gut-lung interactions, gut microbiota dysbiosis and metabolites translocation have been shown to correlate with the severity of emphysema in rats [40]. The importance of rumen microbiota has garnered increasing attention in recent years. Some researchers have elucidated host-microbiota interactions in bovine rumen methanogenesis [41]. Some researchers have also demonstrated that *Bifidobacterium* improves host growth phenotypes by modulating microbial functions and host metabolism [42]. These findings suggest that investigating the alterations in the rumen microbiota of sheep affected by MPS, as well as its relationship with the lung microbiota, may provide insights into the mechanisms by which MPS impacts sheep productivity.

The lung, previously considered a sterile environment, is now recognized to harbor a complex microbiota intrinsically linked to pulmonary pathophysiological processes and lung architecture [43,44]. For instance, a previous study found that the lung microbiota is associated with the development and clinical outcomes of idiopathic pulmonary fibrosis (IPF) [45]. Similarly, significant alterations in the lung microbiota have been observed in patients with COPD [17,18,46,47]. In the present study, we observed significant shifts in the β-diversity of the lung microbiota in sheep with MPS, while alterations in the rumen microbiota were minimal. This muted response within the rumen microbial communities may be attributable to their inherent complexity and resilience. Although multiple *Mycoplasma* species were detected within the lungs of diseased sheep, *M. ovipneumoniae* exhibited consistent presence across all affected animals, confirming its primary pathogenic role in MPS. Given that current literature primarily reports microbial translocation from the gut to extra-intestinal organs via a compromised intestinal barrier [48], and not vice versa, this suggests that microbiota may translocate from the lungs to the rumen in severely affected sheep, potentially through hematogenous dissemination or via the respiratory-digestive tract. Anatomically, the rumen represents the first compartment of the ruminant stomach encountered by ingested material. This anatomical feature, coupled with the rumination process, markedly increases the probability of pulmonary microbiota translocation to the rumen via the respiratory-digestive route. Subsequent analysis of microbial community composition revealed that at the phylum level, the lung microbiota of healthy Hu sheep was primarily composed of *Bacteroidota*, *Proteobacteria*, and *Firmicutes*. Notably, this composition closely mirrored that of the rumen microbiota, potentially reflecting the anatomical and physiological connection between these two compartments. Furthermore, the observed increase in the abundance of *Fusobacteriota* in the lungs of Hu sheep with MPS is consistent with previous studies [21]. At the genus level, a marked increase in *Mycoplasma* and *Pasteurella* was observed in the lungs of diseased Hu sheep, further corroborating that *Mycoplasma* infection can predispose the lung to *Pasteurella* infection [6]. *Pasteurella*, a common animal pathogen, is known to cause rare opportunistic infections [49], suggesting that *Mycoplasma* infection creates favorable conditions for *Pasteurella* colonization, thereby exacerbating the pathological processes in the lungs. Interestingly, a concurrent, significant reduction in the relative abundance of *Succinivibrionaceae_UCG-001* was observed in both the lung and rumen. This parallel decrease provides compelling evidence for inter-compartmental microbial communication. A marked decrease in the abundance of *Prevotella_7* was also observed in the lungs of diseased sheep. Previous studies have indicated the presence of inhaled rumen-derived microbes in the lungs [50], suggesting that MPS may impair respiratory function in sheep, thereby reducing the inhalation of rumen microbiota.

Another interesting finding of this study is the detection of low abundances of *M. ovipneumoniae* in the lungs of seven clinically healthy, asymptomatic sheep. This observation aligns with prior reports [21], supporting the existence of a subclinical carrier state within sheep populations. Despite the absence of overt disease manifestations, these carriers may serve as persistent reservoirs of the pathogen. The presence of such carriers holds substantial implications for disease control and epidemiology. They likely play a pivotal role in transmitting *M. ovipneumoniae* to susceptible individuals, thereby sustaining pathogen circulation within and between sheep flocks. Studies have shown that long-term carriage of *M. ovipneumoniae* by adult females is a source of exposure for naive juveniles [51]. Moreover, this silent colonization elevates the overall risk of future outbreaks. For example, high stocking densities and low ventilation rates in intensive lamb-rearing operations [52], movement of live animals [53], and variations in eco-climate and seasons are all important factors that can induce the disease [54]. Future longitudinal studies should involve long-term tracking of these carrier animals to better elucidate the dynamics of pathogen shedding and the triggering factors underlying the progression to clinical disease.

Differential microbial analysis revealed a distinct lung microbiota profile, characterized by the presence of *Pasteurella*, *Mycoplasma*, *Mannheimia*, *Filobacterium*, *Bacteroides*, and *Fusobacterium*. This observation is highly consistent with previous findings [21]. Further analysis at the species level identified the presence of common pathogens, including *P. multocida subsp. gallicida*, *Mesomycoplasma ovipneumoniae*, *Mycoplasmopsis bovigenitalium*, *Mycoplasmopsis arginini*, and *Mannheimia glucosida*. However, since the reliability of the results of 16S rRNA sequencing analysis at the species level is limited, readers should use this result with caution. These results suggest that the pathological processes involved in MPS are not solely attributable to *M. ovipneumoniae*, but rather involve synergistic interactions among multiple microbial species. This aligns with the known propensity of *M. ovipneumoniae* to predispose hosts to secondary infections [55]. To investigate the relationship between the differential lung and rumen microbiota and productivity in Hu sheep, correlation analyses were conducted. We found a strong positive correlation between pulmonary *Mycoplasma* and *Mannheimia*, and both taxa were significantly associated with higher abundances of ruminal *Prevotellaceae_YAB2003_group* in diseased sheep. Additionally, in the rumen of diseased sheep, *Bifidobacterium*, which was significantly enriched, showed a strong positive correlation with both *Prevotellaceae_YAB2003_group* and *Mannheimia*. *Mannheimia* is a well-known respiratory pathogen that induces pulmonary inflammation and has been reported in cattle, sheep, and goats [56,57,58]. *Bifidobacterium*, on the other hand, is known for its role in modulating host immune responses [59]. These findings suggest that *Mannheimia* may contribute to pulmonary inflammation, and that immune-related factors or microbial metabolites might be transmitted via the rumen-lung axis to the rumen, where they promote the proliferation of *Bifidobacterium*, potentially as a compensatory mechanism to modulate the host immune response. Furthermore, a strong positive correlation was observed between *Pasteurella* and *Succinivibrionaceae_UCG-002*. Conversely, both *Pasteurella* and *Succinivibrionaceae_UCG-002* were strongly negatively correlated with ADG, KR, GFE, RG, and RGR in diseased sheep. Separately, *Succinivibrionaceae_UCG-002* has been implicated in fatty acid metabolism [28]. The observed increase in *Succinivibrionaceae_UCG-002* abundance, potentially driven by *Pasteurella* infection, may further exacerbate this scenario by dysregulating ruminal fatty acid metabolism, contributing to the observed reductions in growth performance and feed efficiency. Interestingly, a highly significant positive correlation was observed between ruminal and pulmonary *Succinivibrionaceae_UCG-001*, further suggesting a potential interplay between the lung and rumen microbial communities. Moreover, *Succinivibrionaceae_UCG-001* levels in both the rumen and pulmonary compartments of diseased sheep were significantly positively correlated with ADFI. *Succinivibrionaceae_UCG-001* is predominantly found in the rumen, where it plays a crucial role in succinate production. Succinate, in turn, is a precursor for propionate, a major energy source for the host [60]. Furthermore, *Succinivibrionaceae_UCG-001* has been negatively correlated with inflammatory cytokines IL-1β and TNFα [61] and positively correlated with growth rate and metabolites associated with high average daily gain (HADG), including propionate, butyrate, maltose, and certain amino acids in young goats [62]. Based on these findings, we think that MPS may lead to reduced appetite and subsequently decreased ADFI, which in turn results in a lower availability of fermentable substrates required for the growth of *Succinivibrionaceae_UCG-001* in the rumen. The decline in nutrient availability may contribute to the reduced abundance of this taxon in the rumen. Consequently, the diminished ruminal reservoir of *Succinivibrionaceae_UCG-001* may lead to a decreased likelihood of its translocation to the lungs via the proposed rumen-lung axis, potentially explaining its reduced presence in pulmonary microbiota under disease conditions. Moreover, the synergistic action of MPS likely triggers a pulmonary inflammatory response, resulting in the production of pro-inflammatory cytokines. These cytokines can enter systemic circulation, reaching the rumen and potentially perturbing ruminal metabolism. This could contribute to the observed reduction in ruminal *Succinivibrionaceae_UCG-001* abundance. Via a proposed rumen-lung axis, a diminished ruminal *Succinivibrionaceae_UCG-001* population could also lead to reduced translocation of this microbe to the lungs. Focusing on the species level, *P. multocida* subsp. *gallicida* exhibited a highly significant positive correlation with the relative weight of lung in diseased sheep. This strong association, combined with the negative correlations between *P. multocida* subsp. *gallicida* and growth/feed efficiency parameters, points to *P. multocida* subsp. *gallicida* as a key bacterial strain contributing to the detrimental effects of MPS on Hu sheep productivity. Importantly, our findings suggest that regulating rumen microbiota could enhance the clinical treatment of MPS. For one, the observed interplay between rumen and lung microbiota implies that modulating rumen communities may promote the reconstruction of lung microbiota. For another, given the involvement of rumen microbiota in mediating MPS-induced impairments to host growth performance, targeted regulation of these microbial communities might also mitigate such growth-related deficits. Collectively, these insights indicate that rumen microbiota modulation could facilitate the host’s recovery from MPS.

Despite the intriguing findings presented in this study, several limitations should be acknowledged. First, the relatively small sample size and the inclusion of only a single sheep breed limit the generalizability of our results. Future studies should aim to increase the cohort size and incorporate animals from diverse geographical regions, breeds, and age groups to validate the universality of the observed microbiome alterations. Moreover, this study did not account for host genetic background or immune status, both of which are known to influence microbial community composition and may confound associations between microbial dysbiosis and disease phenotype. Integrating host genomic and immunological data will be essential in future investigations to enhance the interpretability and translational relevance of microbiome findings. In addition, experimental validation using gnotobiotic animal models or microbiota transplantation could provide critical evidence to establish causal links between microbial shifts and disease progression. Finally, the absence of metabolomic analyses limits our ability to infer the functional consequences of microbial alterations. Future research integrating multi-omics approaches, including metagenomics, metabolomics, and host transcriptomics, will be instrumental in elucidating the mechanistic roles of key microbial taxa and their interactions with the host during disease development.

## 5. Conclusions

In conclusion, this study demonstrates that MPS significantly impairs sheep productivity and induces pronounced alterations in pulmonary microbiota, while exerting a comparatively limited effect on the rumen microbial community. Further analysis suggests a potential crosstalk between the lung and rumen microbiota in diseased animals, which may contribute to the observed decline in performance. Notably, *Pasteurella multocida* subsp. *gallicida* emerged as a potential key pathogen linked to reduced feed efficiency. This research provides novel insights into MPS pathogenesis and lays the groundwork for developing more effective prevention and control strategies.

## Data Availability

The raw sequence data reported in this paper have been deposited in the Genome Sequence Archive in the National Genomics Data Center, China National Center for Bioinformation/Beijing Institute of Genomics, Chinese Academy of Sciences (GSA: CRA028127), which are publicly accessible at https://ngdc.cncb.ac.cn/gsa (accessed on 24 July 2025).

## Supplementary Materials

The following supporting information can be downloaded at: https://www.mdpi.com/article/10.3390/vetsci12080741/s1, Figure S1: Sequencing depth was assessed with dilution curves; Figure S2: Heatmap of the correlation analysis between differential microbiota in the lung and rumen and growth performance and feed efficiency in diseased sheep. Table S1: Diet information for animal experiments (air-dry basis). Table S2: ASVs belonging to the genus *Mycoplasma*. Table S3: Unique ASVs in diseased lungs.

## Abbreviations

The following abbreviations are used in this manuscript:

MPS	Mycoplasmal pneumonia of sheep
BW	Body weight
ADG	Average daily gain
ADFI	Average daily feed intake
MBW	Metabolic body weight
FCR	Feed conversion ratio
KR	Kleiber ratio
GFE	Gross feed efficiency
DMIMBW	Dry matter intake per unit metabolic body weight
RG	Residual gain
RGR	Relative growth rate
ASV	Amplicon sequence variant
NP	Neutrophil percentage
CCG	Chest circumference gain
