# Supplementary material for: Multidimensional Phenotypic and Microbiome Studies Uncover an Association Between Reduced Feed Efficiency in Sheep During Mycoplasmal Pneumonia and Microbial Crosstalk Within the Rumen-Lung Axis
Source: Vet Sci. 2025 Aug 7;12(8):741. doi: 10.3390/vetsci12080741 (PMC12390611; doi:10.3390/vetsci12080741)
Supplement: Supplementary file 1 [file vetsci-12-00741-s001.zip › vetsci-3740923-supplementary-english.pdf]

Multidimensional Phenotypic and Microbiome Studies Uncover an Association Between Reduced Feed Efficiency in Sheep During Mycoplasmal Pneumonia and Microbial Crosstalk Within the Rumen-Lung Axis

Lianjun Feng [1,†], Yukun Zhang [1,†], Xiaoxue Zhang [2], Fadi Li [1], Kai Huang [1], Deyin Zhang [1], Zongwu Ma [1],
Chengqi Yan [1], Qi Zhang [1], Mengru Pu [1], Ziyue Xiao [1], Lei Gao [1], Changchun Lin [2], Weiwei Wu [3], Weimin Wang [1] and Huibin Tian [1,*]

Supplementary material

Table S1

Diet information for animal experiments (air-dry basis).

Ingredient composition (% as fed)		Chemical composition	
Corn	32.5	Dry matter (DM) [%]	88.78
Corn germ meal	18	Crude protein (CP) [%]	13.09
Corn stalks	12	Digestible energy [MJ/kg]	11.11
Corn hulls	11.2	Neutral detergent fiber (NDF) [%]	27.08
Corn cob	8	Acid detergent fiber (ADF) [%]	13.99
Soybean meal	5	Crude fiber (CF) [%]	9.78
Cotton meal	5		
Molasses	3.3		
Bentonite	1.5		
Baking soda	1		
Stone powder	0.8		
Expanded Urea	0.5		
Premix	0.5		

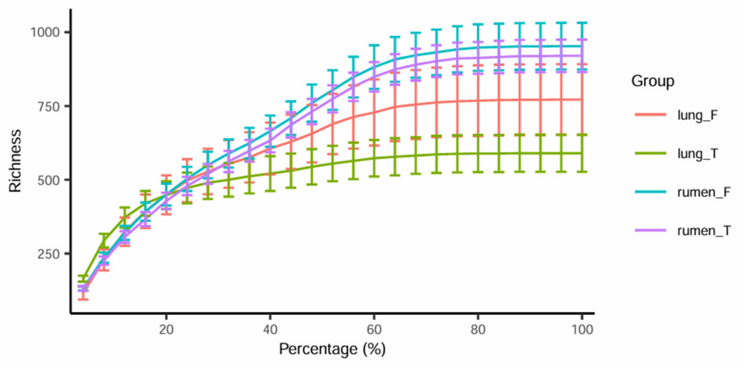

Figure. S1. Sequencing depth was assessed with dilution curves.

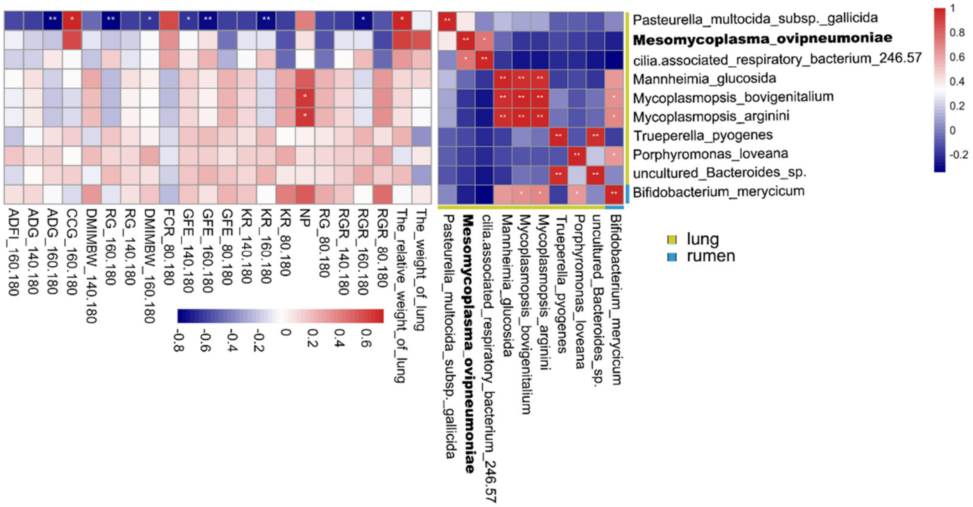

Figure. S2. Heatmap of the correlation analysis between differential microbiota in the lung and rumen and growth performance and feed efficiency in diseased sheep. The method of correlation analysis uses Pearson; $*P < 0.05$, $**P < 0.01$.
